# Adjunctive Effects of a 2% Zinc Citrate Toothpaste on Plaque and Oral Microbiota in Orthodontic Patients: A Randomized Controlled Trial

**DOI:** 10.1155/ijod/9264740

**Published:** 2026-09-28

**Authors:** Thi Thanh Ngoc Nguyen, Trung Chanh Le, Nguyen Quan Pham, Tin Hien Pham, Thi Quynh Lan Ngo

**Affiliations:** ^1^ Department of Periodontics, National Hospital of Odonto-Stomatology, Ho Chi Minh City, Vietnam; ^2^ Department of Dental Implantology, National Hospital of Odonto-Stomatology, Ho Chi Minh City, Vietnam; ^3^ Faculty of Odonto-Stomatology, Hong Bang International University, Ho Chi Minh City, Vietnam; ^4^ Faculty of Dentistry, University of Medicine and Pharmacy at Ho Chi Minh City, Ho Chi Minh City, Vietnam, hcmut.edu.vn

**Keywords:** gingivitis, oral hygiene, orthodontics, zinc citrate

## Abstract

**Introduction:**

Zinc citrate trihydrate (ZCT) has antibacterial and anti‐inflammatory properties and is widely used in oral‐care products. However, evidence regarding its effectiveness in orthodontic patients remains limited. This study aimed to evaluate the effects of a toothpaste containing 2% ZCT on gingival inflammation, plaque accumulation, and plaque‐associated bacterial load in patients wearing fixed orthodontic appliances.

**Methods:**

A randomized controlled clinical trial was conducted among 102 patients with plaque‐induced gingivitis undergoing fixed orthodontic treatment. Participants were randomly assigned to use either a toothpaste containing 2% ZCT (test group) or a conventional toothpaste without ZCT (control group). The gingival index (GI) and Modified Quigley–Hein Plaque Index (MQHPI) were recorded at baseline, after 1 week, and after 4 weeks. Supragingival plaque samples were collected at baseline and after 4 weeks and analyzed using real‐time polymerase chain reaction to quantify selected plaque‐associated bacterial species. Linear mixed‐effects models were used to evaluate the effects of time, treatment, and their interaction on GI and MQHPI.

**Results:**

Exploratory time‐specific comparisons at 4 weeks showed lower GI and MQHPI and lower loads of *Fusobacterium nucleatum* (Fn), *Tannerella forsythia* (Tf), and *Solobacterium moorei* (Sm) in the ZCT group. However, the longitudinal mixed‐effects analyses showed no significant treatment‐by‐time interaction for either the clinical outcomes or the quantitatively assessable bacterial species.

**Conclusions:**

Both groups showed clinical improvement over time. However, the longitudinal analyses did not demonstrate an additional clinical or microbiological effect attributable to the 2% ZCT toothpaste during the 4‐week study period.

## 1. Introduction

Gingivitis is the most prevalent periodontal condition both in Vietnam and worldwide [1]. For individuals undergoing fixed orthodontic treatment, maintaining adequate oral hygiene can be particularly challenging. Fixed orthodontic appliances create additional plaque‐retentive areas, thereby complicating daily oral hygiene practices and increasing the risk of gingival inflammation and other periodontal diseases [2]. Previous studies have reported a high prevalence of gingivitis among orthodontic patients [3, 4]. For instance, Ramamurthy and Gopalasamy [4] reported that ~76% of patients undergoing orthodontic treatment presented with gingivitis. These findings highlight the importance of effective plaque control strategies for patients wearing fixed orthodontic appliances.

To address these challenges, various oral hygiene devices and chemical agents have been developed to improve plaque control in both the general population and orthodontic patients. Dental plaque is now recognized as a complex microbial biofilm that plays a central role in the development of gingivitis and periodontal diseases [5]. Among the chemical agents used to control plaque, zinc citrate trihydrate (ZCT) has attracted considerable interest because of its antibacterial and anti‐inflammatory properties [6, 7]. Zinc citrate is commonly incorporated into oral care formulations such as toothpastes, mouthrinses, and chewing gums. The United States Food and Drug Administration [8] permits the use of zinc citrate in oral care products at concentrations ranging from 0.5% to 2%. Previous studies have suggested that zinc‐containing dentifrices may reduce dental plaque accumulation and bacterial load, thereby improving gingival health. For example, Barnes et al. [9] reported that a toothpaste containing zinc citrate significantly reduced plaque formation compared with a control dentifrice. Furthermore, Sreenivasan et al. [10] demonstrated that a zinc citrate dentifrice significantly reduced bacterial counts on several oral surfaces. Similarly, Adams et al. [11] showed that a dentifrice containing zinc citrate combined with triclosan produced greater inhibition of plaque formation than a triclosan‐only formulation. Given the concerns regarding the use of triclosan in consumer products, it is crucial to evaluate zinc‐based formulations free from such controversial agents. Therefore, the test formulation in the present study relies solely on 2% ZCT as the active antibacterial agent without triclosan or other active compounds.

However, most previous clinical trials have been conducted in general populations rather than in orthodontic patients. Moreover, relatively few investigations have simultaneously evaluated both clinical periodontal indices and microbiological changes associated with the use of zinc‐containing toothpastes in patients wearing fixed orthodontic appliances. Therefore, the aim of this randomized, parallel‐group, controlled clinical trial was to evaluate the effectiveness of a toothpaste containing 2% ZCT in reducing gingival inflammation, dental plaque accumulation, and plaque‐associated bacterial load compared with a conventional toothpaste without ZCT in this patient population.

## 2. Materials and Methods

### 2.1. Study Design and Sample Size

This study was designed as a randomized, parallel‐group, controlled clinical trial. Participants were randomly assigned to the study groups in a 1:1 ratio using a simple randomization procedure based on drawing lots. Treatment groups were initially coded as “0” and “1.” After enrollment, each participant personally drew a folded lot containing one of these coded assignments under the supervision of a research assistant who was not involved in treatment delivery, clinical examination, or outcome assessment. The selected code was recorded and linked to the corresponding study toothpaste only at the subsequent dispensing step. The two groups were (1) the ZCT group, who used a toothpaste containing 2% ZCT, and (2) the control group, who used a conventional toothpaste without ZCT. The control toothpaste was a standard daily‐use fluoride dentifrice (containing 1450 ppm fluoride). The base formulations of both toothpastes (including abrasives, humectants, and fluoride content) were completely identical, except for the addition of 2% ZCT in the test dentifrice. The sample size was calculated based on a previous study conducted in orthodontic patients by Boyd and Chun [2]. Assuming a two‐sided significance level (*α*) of 0.05 and a statistical power of 80% to detect a difference in mean plaque index between the control group (mean: 1.06, SD: 0.61) and the test group (mean: 0.68, SD: 0.61), a minimum of 41 participants per group was required. The study ultimately enrolled 102 participants to compensate for potential attrition, all of whom completed follow‐up and were included in the analyses. Since an exact sample size calculation for microbiological parameters could not be performed due to the lack of similar reference studies in this specific patient population, previous general periodontal studies were conducted on a similar sample size to detect quantitative differences in bacterial loads [12, 13]. Therefore, our sample size of 51 participants per group for the microbiological analysis.

### 2.2. Patient Selection

A total of 102 patients aged 18 years or older undergoing fixed orthodontic treatment were recruited from the Department of Periodontics at the National Hospital of Odonto‐Stomatology, Ho Chi Minh City, Vietnam, between July 2024 and October 2024.

Participants were eligible if they met the following inclusion criteria:

• The presence of labial metal brackets and archwires in both dental arches.
• At least 20 natural teeth are present, including at least five teeth in each quadrant (excluding third molars).
• The diagnosis of moderate to severe plaque‐induced gingivitis affecting at least one tooth in each quadrant without subgingival calculus.

Participants were excluded if they met any of the following criteria:

• Diagnosis of periodontitis.
• The presence of acute gingival or oral mucosal lesions.
• Current smoking or a history of smoking within the previous 5 years.
• Uncontrolled systemic diseases.
• The use of antibiotics or anti‐inflammatory medications within 4 weeks before the first visit.
• Pregnancy or breastfeeding.
• Known allergy to zinc citrate.

### 2.3. Study Procedures

This randomized controlled trial was reported according to the CONSORT 2010 guidelines. The overall study procedures are illustrated in the flowchart. After enrollment and baseline assessment, participants were assigned to one of the two study groups and were instructed to brush their teeth twice daily using the assigned toothpaste throughout the study period. Clinical examinations were performed at three time points: baseline, 1 week, and 4 weeks (Figure 1).

**Figure 1:**
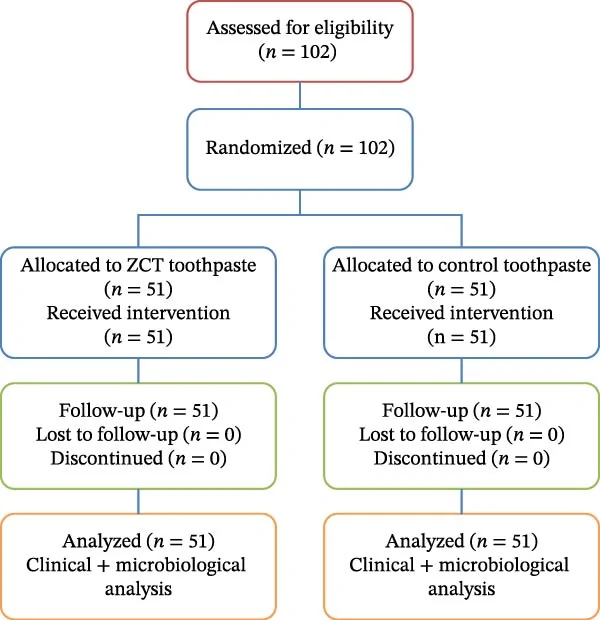
CONSORT 2010 flow diagram.

### 2.4. Clinical Outcomes

Clinical periodontal parameters were assessed using the following indices: gingival index (GI) according to Löe and Silness [1], Modified Quigley–Hein Plaque Index (MQHPI), according to Turesky et al. [14] modification. Both indices were recorded at baseline, after 1 week, and after 4 weeks.

### 2.5. Microbiological Outcomes

Supragingival plaque samples were collected at baseline and after 4 weeks. To ensure adequate plaque accumulation for reliable sampling, all participants were instructed to refrain from any oral hygiene procedures (including brushing, flossing, and mouthrinsing) for 12 h (overnight) prior to their scheduled clinical assessment and plaque collection appointments. A targeted sampling strategy was used to obtain an adequate amount of supragingival plaque for microbiological analysis, given that plaque accumulation around fixed orthodontic appliances may vary considerably among teeth and sites. In each quadrant, the three teeth showing the greatest visible plaque accumulation during the clinical plaque assessment were selected for sampling, resulting in a total of 12 sampled teeth per participant. The same predefined selection procedure was applied at baseline and at the 4‐week assessment. Supragingival plaque was collected from the buccal and lingual surfaces of the selected teeth using a sterile Gracey curette. Plaque collected from all 12 teeth was pooled into a single 1.5‐mL Eppendorf tube containing phosphate‐buffered saline (PBS) for each participant at each sampling time point. Accordingly, the participant, rather than the individual tooth or sampling site, constituted the unit of microbiological analysis. Samples were transported on ice to the microbiology laboratory within 4 h and stored at −70°C until analysis (Figure 2).

**Figure 2:**
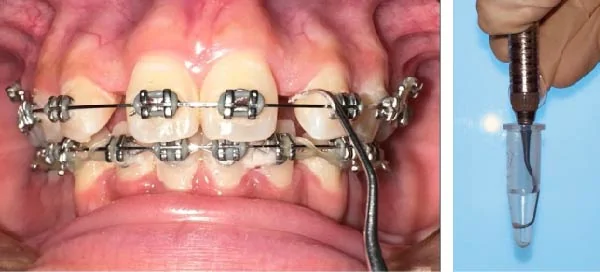
Plaque sampling.

Plaque samples were processed using a laboratory protocol similar to that previously described by Nguyen et al. [15]. The samples suspended in PBS were centrifuged at 9300 rpm for 3 min. After removal of PBS, the resulting pellet was resuspended in 200 µL Tris‐EDTA buffer for DNA extraction. Quantitative real‐time PCR was performed using a CFX96 Real‐Time PCR System (Bio‐Rad, Hercules, CA, USA) with species‐specific assays to quantify *Aggregatibacter actinomycetemcomitans* (Aa), *Fusobacterium nucleatum* (Fn), *Porphyromonas gingivalis* (Pg), *Treponema denticola* (Td), *Tannerella forsythia* (Tf), and *Solobacterium moorei* (Sm). Bacterial loads were expressed as copies/mL.

### 2.6. Bias Control and Blinding

To preserve examiner and participant blinding and to separate treatment allocation from outcome assessment, the research team was divided into two independent subteams. Team A (one dentist and one nurse) performed clinical examinations and plaque sample collections, remained completely blinded to the treatment allocation, and had no access to the patient codes or group assignments throughout the study. Prior to the trial, the single clinical examiner (from Team A) underwent formal calibration training. Intraexaminer reliability was assessed on 10 nonstudy orthodontic patients, yielding a Kappa coefficient of 0.85 for the GI and 0.82 for the MQHPI, demonstrating excellent reproducibility. Team B (one dentist and two other nurses) was responsible for participant allocation, keeping the randomization sequence and patient codes strictly confidential, dispensing the assigned toothpaste, and providing oral hygiene instructions. The toothpaste tubes were identical in design to ensure that participants remained blinded to their group assignment. To maintain compliance, nurses in Team B were tasked with providing reminders regarding the instructed brushing method and assigned dentifrice through phone calls or text messages, conducted at 3‐day intervals. All plaque samples were coded before being sent to the microbiology laboratory, ensuring that laboratory personnel were blinded to group allocation. The examiner who assessed the gingival status and plaque accumulation did not have access to treatment allocation data.

### 2.7. Data Management and Statistical Analysis

All statistical analyses were performed using SPSS version 26.0. Prior to conducting parametric tests, the normality of the data distribution and residuals for clinical variables was assessed and confirmed using skewness and kurtosis values. For clinical outcomes, linear mixed‐effects models were used as the primary inferential analyses. Separate models were fitted for GI and MQHPI, with time (baseline, 1 week, and 4 weeks), treatment group (ZCT vs. control), and the treatment‐by‐time interaction included as fixed effects. A participant‐level random intercept was included to account for within‐participant correlation arising from repeated measurements. Time‐specific between‐group comparisons were performed only as exploratory analyses and were not used to infer an additional longitudinal treatment effect.

To stabilize variance and normalize the highly skewed distribution of the microbiological data, all absolute bacterial copy numbers obtained from real‐time PCR were log‐transformed (log_10_ copies/mL) prior to statistical analysis. Similar linear mixed‐effects models were fitted for microbiological data, with time (baseline and 4 weeks), treatment group (ZCT vs. control), and the treatment‐by‐time interaction included as fixed effects. The *p*‐values for the treatment‐by‐time interaction were adjusted using the Holm method. For microbiological outcomes, the Wilcoxon signed‐rank test was used to compare bacterial loads between baseline and 4 weeks within each group, while the Mann–Whitney *U* test was used to compare bacterial loads between groups at baseline and at 4 weeks for descriptive and exploratory time‐specific analyses. These quantitative nonparametric analyses were applied only to bacterial species with sufficient detection rates. A *p*‐value < 0.05 was considered statistically significant in this study.

### 2.8. Ethical Clearance

The toothpaste containing 2% ZCT used in this study has been approved for use in oral care products by the Vietnamese Ministry of Health. All work involving human subjects was conducted in accordance with the Declaration of Helsinki and its subsequent amendments. The study protocol was approved by the Institutional Review Board (Biomedical Research Ethics Committee) of the National Hospital of Odonto‐Stomatology, Ho Chi Minh City, Vietnam, under Decision Number 682/QD‐RHMTW. The trial was not prospectively registered. The experiment was conducted with the participants’ understanding and written informed consent prior to participation. Adverse events and oral soft‐tissue reactions were assessed at each follow‐up visit.

## 3. Results

### 3.1. Baseline Characteristics

A total of 102 participants were enrolled in the study and evenly allocated to the two groups (51 participants in each group). In the ZCT group, there were 8 males (15.7%) and 43 females (84.3%), with a mean age of 23.49 ± 4.98 years. In the control group, there were 9 (17.6%) males and 42 females (82.4%), with a mean age of 24.53 ± 5.33 years (Table 1).

**Table 1:** Baseline participant characteristics.

Characteristic	Control (*n* = 51)	ZCT 2% (*n* = 51)	*p*‐Value
Age (years), mean ± SD	24.53 ± 5.33	23.49 ± 4.98	0.31
Sex *n* (%)			1.000
Male	9 (17.6)	8 (15.7)	
Female	42 (82.4)	43 (84.3)	
Baseline GI, mean ± SD	2.16 ± 0.39	2.19 ± 0.35	0.65
Baseline MQHPI, mean ± SD	3.07 ± 0.43	3.14 ± 0.55	0.45

No statistically significant differences were observed between the two groups in terms of age, sex distribution, baseline GI, or baseline MQHPI (*p* > 0.05), indicating that the baseline characteristics of the two groups were comparable. No adverse events, soft‐tissue irritation, or allergic reactions were observed or reported during the study.

### 3.2. Changes in GI and Plaque Index

Both groups demonstrated significant reductions in GI and MQHPI at 1 week and 4 weeks compared with baseline values (*p* < 0.001). At the 1‐week follow‐up, no statistically significant differences in GI or MQHPI were observed between the ZCT and control groups (*p* > 0.05). However, at the 4‐week assessment, time‐specific between‐group comparisons showed significantly lower GI and MQHPI values in the ZCT group than in the control group (*p* < 0.05) (Table 2, Figure 3). The mean difference between the ZCT and control groups (ZCT–control) was −0.13 for GI (95% CI: from −0.244 to −0.016) and −0.22 for MQHPI (95% CI: from −0.375 to −0.065). These comparisons were considered exploratory and should be interpreted in conjunction with the primary longitudinal mixed‐effects analysis presented in Section 3.4.

**Figure 3:**
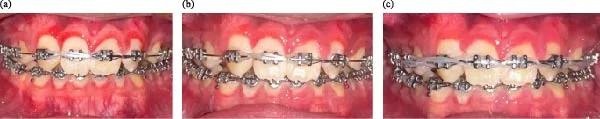
Clinical appearance of gingival tissues in patients using toothpaste containing 2% zinc citrate trihydrate at baseline (a), after 1 week (b), and after 4 weeks (c).

**Table 2:** GI and MQHPI recorded at baseline, 1 week, and 4 weeks.

Outcome	Group	Baseline	1 week	4 weeks
GI	Control (*n* = 51)	2.16 ± 0.39	1.36 ± 0.32	1.58 ± 0.30
	ZCT 2% (*n* = 51)	2.19 ± 0.35	1.41 ± 0.32	1.45 ± 0.28
	*p*	0.65	0.40	<0.05 ^∗^

MQHPI	Control (*n* = 51)	3.07 ± 0.43	2.03 ± 0.37	2.33 ± 0.38
	ZCT 2% (*n* = 51)	3.14 ± 0.55	2.07 ± 0.40	2.11 ± 0.41
	*p*	0.45	0.68	<0.05 ^∗^

^∗^ *p* < 0.05.

### 3.3. Changes in Plaque‐Associated Bacterial Load

Microbiological analysis revealed that *Aa* and *Td* were rarely detected in supragingival plaque samples at baseline and at the 4‐week assessment in both groups (Table 3). At the 4‐week follow‐up, significant differences were observed between groups for several bacterial species. The loads of Fn, Tf, and Sm were significantly lower in the ZCT group compared with the control group (*p* < 0.05). In contrast, no statistically significant difference was observed for Pg between the two groups (*p* > 0.05). These time‐specific between‐group differences were considered exploratory and were interpreted in conjunction with the longitudinal mixed‐effects analyses presented in Section 3.5.

**Table 3:** Supragingival plaque bacterial load at baseline and 4 weeks.

Bacteria	Group	Baseline (log_10_ copies/mL)	4 weeks (log_10_ copies/mL)
Aa	Control (*n* = 51)	Rarely detected	Rarely detected
	ZCT (*n* = 51)	Rarely detected	Rarely detected

Fn	Control (*n* = 51)	6.75 (6.35–7.06)	6.79 (6.45–7.22)
	ZCT (*n* = 51)	6.92 (6.49–7.11)	5.29 (4.40–6.30)
	*p*	0.36	**0.001 ^∗^**

Pg	Control (*n* = 51)	2.01 (0–4.34)	2.05 (0–3.78)
	ZCT (*n* = 51)	2.95 (0–4.11)	3.10 (0–4.63)
	*p*	0.56	0.31

Td	Control (*n* = 51)	Rarely detected	Rarely detected
	ZCT (*n* = 51)	Rarely detected	Rarely detected

Tf	Control (*n* = 51)	3.67 (0–4.81)	3.50 (0–4.29)
	ZCT (*n* = 51)	3.35 (2.14–4.29)	2.37 (2.35–5.03)
	*p*	0.59	**0.04 ^∗^**

Sm	Control (*n* = 51)	4.38 (3.65–5.29)	4.24 (3.06–4.88)
	ZCT (*n* = 51)	4.03 (3.11–4.78)	4.10 (0–3.73)
	*p*	0.25	**0.001 ^∗^**

*Note:* The bold values indicate statistically significant differences between the comparison groups.

^∗^ *p* < 0.05.

### 3.4. Effects of Time, Treatment, and Their Interaction on GI and MQHPI

The primary longitudinal analyses using linear mixed‐effects models confirmed a significant effect of time on both GI (*F* = 327.870, *p*  < 0.001) and MQHPI (*F* = 753.608, *p*  < 0.001). However, neither the treatment effect (GI: *p* = 0.689; MQHPI: *p* = 0.392) nor the treatment‐by‐time interaction (GI: *p* = 0.833; MQHPI: *p* = 0.658) reached statistical significance (Table 4).

**Table 4:** Linear mixed‐effects model analysis of GI and MQHPI.

Fixed effect	GI, *F* (df)	*p*‐Value	MQHPI, *F* (df)	*p*‐Value
Time	327.870 (2100)	<0.001	753.608 (2100)	**<0.001 ^∗^**
Treatment	0.162 (1100)	0.689	0.738 (1100)	0.392
Time × treatment	0.183 (2100)	0.833	0.420 (2100)	0.658

*Note:* The bold values indicate statistically significant differences between the comparison groups.

^∗^ *p* < 0.05.

### 3.5. Effects of Time, Treatment, and Their Interaction on Bacterial Load

The treatment‐by‐time interactions were not statistically significant for Fn (*F* = 2.535, *p* = 0.115), Pg (*F* = 0.028, *p* = 0.868), Sm (*F* = 0.060, *p* = 0.808), or Tf (*F* = 3.276, *p* = 0.073). The corresponding Holm‐adjusted *p*‐values were 0.345 for Fn, 1.000 for Pg, 1.000 for Sm, and 0.292 for Tf. None was statistically significant (Table 5). Accordingly, the previously reported time‐specific differences at 4 weeks are now considered exploratory and are not interpreted as evidence of an additional longitudinal microbiological effect of ZCT.

**Table 5:** Linear mixed‐effects model analysis of plaque‐associated bacterial loads.

Fixed effect	Fn, *F* (df)	*p*‐Value	Pg, *F* (df)	*p*‐Value	Sm, *F* (df)	*p*‐Value	Tf, *F* (df)	*p*‐Value
Time	0.022 (1100)	0.883	0.013 (1100)	0.909	1.251 (1100)	0.266	2.149 (1100)	0.146
Treatment	0.758 (1100)	0.386	0.468 (1100)	0.495	0.488 (1100)	0.486	0.083 (1100)	0.774
Time × treatment	2.535 (1100)	0.115	0.028 (1100)	0.868	0.060 (1100)	0.808	3.276 (1100)	0.073

## 4. Discussion

This randomized controlled clinical trial demonstrated significant improvements in gingival health and plaque control over time in both groups of patients undergoing fixed orthodontic treatment. However, the primary longitudinal analyses showed no significant treatment effect or treatment‐by‐time interaction for GI or MQHPI. Similarly, the longitudinal microbiological analyses showed no significant treatment‐by‐time interaction for any of the quantitatively assessable bacterial species. Although exploratory time‐specific comparisons at 4 weeks showed between‐group differences in selected clinical and microbiological outcomes, these findings were not supported by the longitudinal interaction analyses and should therefore be interpreted cautiously.

Previous clinical investigations have reported beneficial effects of zinc‐containing dentifrices (Barnes et al. [9] and Adams et al. [11]). Direct comparisons should be made cautiously because of differences in study populations, dentifrice formulations, study durations, and microbiological assessment methods. Most previous investigations were conducted in the general healthy population, often utilizing formulations combined with now‐restricted agents like triclosan. Our study extends the available evidence by evaluating a triclosan‐free 2% ZCT formulation specifically in patients undergoing fixed orthodontic treatment. Experimental and microbiological studies have also demonstrated the antimicrobial activity of zinc‐containing formulations [7, 16]. Shukla et al. [16] reported that zinc citrate formulations reduced several oral bacterial species in vitro, suggesting that zinc compounds may interfere with bacterial metabolism and biofilm formation. However, the present longitudinal analyses did not demonstrate a statistically significant differential change in the quantitatively assessable bacterial species between the ZCT and control groups over the 4‐week study period. Therefore, while the exploratory time‐specific findings may be considered in the context of the previously reported antimicrobial properties of zinc, they do not provide confirmatory evidence of an additional longitudinal microbiological effect of ZCT.

Although exploratory time‐specific comparisons showed significantly lower GI and MQHPI values in the ZCT group at the 4‐week follow‐up, to better account for the repeated‐measures design, the linear mixed‐effects models, which constituted the primary longitudinal analyses, showed significant changes over time in both outcomes but no significant overall treatment effect or treatment‐by‐time interaction. This indicates that the magnitude and trajectory of clinical improvement were comparable between the ZCT and control groups. The improvements observed in both groups may partly reflect standardized oral hygiene instruction and regular monitoring rather than an additional measurable clinical effect of the zinc citrate dentifrice. However, because daily oral hygiene behavior (such as brushing duration and technique adherence) was not objectively measured during the study, these behavioral improvements remain a plausible but indirect explanation. Previous studies in orthodontic patients have similarly reported reductions in plaque and gingival indices following reinforcement of oral hygiene measures [2, 9].

For the microbiological outcomes, exploratory time‐specific comparisons at 4 weeks showed between‐group differences for the selected bacterial species. However, these comparisons do not directly evaluate whether the changes from baseline differed between treatment groups. In the longitudinal mixed‐effects analyses, no statistically significant treatment‐by‐time interaction was observed for Fn, Pg, Sm, or Tf. Therefore, the present findings do not demonstrate an additional longitudinal microbiological effect attributable to the 2% ZCT toothpaste during the 4‐week observation period. Previous studies have shown that zinc citrate had antimicrobial activity and may inhibit plaque formation and bacterial viability through disruption of bacterial metabolism and biofilm development [6, 11, 16]. Fn and Tf are plaque‐associated bacterial species with established roles in periodontal biofilm ecology and periodontal disease [5, 13]; therefore, the exploratory differences observed for these species may be of biological interest. However, given the absence of significant treatment‐by‐time interactions, these time‐specific findings should not be interpreted as evidence that ZCT produced a sustained or differential reduction in these organisms. Aa and Td were rarely detected and could not be meaningfully evaluated using quantitative longitudinal models. Longer‐term studies specifically designed and powered for microbiological outcomes are needed to determine whether ZCT produces species‐specific changes in the supragingival biofilm.

From a clinical perspective, fixed orthodontic appliances facilitate plaque retention, while the evidence regarding adjunctive chemical plaque control in this population remains limited. In the present study, both groups showed substantial clinical improvement over time; however, the longitudinal analyses did not demonstrate an additional clinical or microbiological effect attributable to the 2% ZCT toothpaste during the 4‐week observation period. These findings emphasize the importance of continued mechanical plaque control and oral hygiene reinforcement in patients undergoing fixed orthodontic treatment. Whether ZCT provides additional microbiological effects over longer periods requires further investigation.

Several limitations of this study should be acknowledged. First, the follow‐up period was relatively short, which limits the ability to assess the long‐term effects of zinc citrate toothpastes on periodontal health. Second, oral hygiene behavior was not objectively monitored on a daily basis (e.g., through diaries or digital sensors), which limits our ability to verify exact home care compliance. The isolated between‐group differences observed at 4 weeks should be regarded as exploratory findings and do not constitute evidence of an additional clinical or microbiological effect of ZCT because the longitudinal treatment‐by‐time interaction was not statistically significant. In addition, the targeted sampling of plaque‐rich sites may not fully represent the whole‐mouth supragingival microbiological profile. Future studies with longer follow‐up periods, larger sample sizes, and additional behavioral assessments are needed to further clarify the long‐term effectiveness of zinc‐containing dentifrices in orthodontic patients.

## 5. Conclusion

Both study groups demonstrated significant improvements in gingival inflammation and plaque accumulation over time. Although exploratory time‐specific comparisons at 4 weeks showed differences in selected clinical and microbiological outcomes, the longitudinal mixed‐effects analyses demonstrated no significant treatment‐by‐time interaction for either the clinical outcomes or the quantitatively assessable plaque‐associated bacterial species.

## Author Contributions

**Thi Thanh Ngoc Nguyen**: conceptualization, investigation, writing – original draft. **Trung Chanh Le** and **Thi Quynh Lan Ngo**: conceptualization, methodology, supervision, writing – review and editing. **Nguyen Quan Pham**: formal analysis, writing – review and editing. **Tin Hien Pham**: investigation, validation.

## Funding

This study was financially supported by a research grant from the Vietnam Odonto‐Stomatology Association (VOSA).

## Disclosure

All authors have read and approved the last version of the manuscript. Le Trung Chanh had full access to all the data in this study and takes complete responsibility for the integrity of the data and the accuracy of the data analysis. The funder had no role in the study design, clinical procedures, data collection and statistical analysis, interpretation of the results, decision to publish, or preparation of the manuscript.

## Ethics Statement

The study protocol was approved by the Institutional Review Board (Biomedical Research Ethics Committee) of the National Hospital of Odonto‐Stomatology, Ho Chi Minh City, Vietnam, under Decision Number 682/QD‐RHMTW.

## Conflicts of Interest

The authors declare no conflicts of interest.

## Data Availability

The data that support the findings of this study are available upon request from the corresponding author. The data are not publicly available due to privacy or ethical restrictions.
